# A droplet digital PCR method for the detection of scale drop disease virus in yellowfin seabream (*Acanthopagrus latus*)

**DOI:** 10.3389/fmicb.2024.1444235

**Published:** 2024-09-25

**Authors:** Bin Yin, Can Mao, Fangzhao Yu, Wangdong Li, Runhong Pan, Wei Feng, Yong Li

**Affiliations:** Zhuhai Modern Agriculture Development Center, Zhuhai, China

**Keywords:** yellowfin seabream (*Acanthopagrus latus*), scale drop disease virus, droplet digital PCR, annealing temperature, clinical detection

## Abstract

In this study, a ddPCR method for the detection of scale drop disease virus (SDDV) in yellowfin seabream (*Acanthopagrus latus*) was established based on Real-time fluorescence quantitative PCR detection methods and principles. The reaction conditions were optimized, and the sensitivity, specificity, accuracy, and reproducibility were assessed. The results showed that threshold line position was determined to be 1900 by the ddPCR method; the optimum annealing temperature for SDDV detection by the ddPCR method was 60°C; the limit of detection was 1.4–1.7 copies/μL; the results of specific detection of other common viruses, except for SDDV specific amplification, were all negative; and the relative standard deviation (RSD) for the reproducibility validation was 0.77%. The samples of yellowfin seabream (*Acanthopagrus latus*) liver, spleen, kidney, heart, intestine, brain, blood, muscle, skin and ascites with three replicates, respectively, were tested using the ddPCR method, and the results were consistent with clinical findings. The ddPCR method established in this study has the advantages of high sensitivity, high specificity, good reproducibility and simple steps for the quantitative detection of SDDV, which could be used for the nucleic acid detection of clinical SDDV samples, and provided a new quantitative method for the diagnosis of yellowfin seabream SDDV in the early stage of pathogenesis.

## 1 Introduction

The yellowfin seabream (*Acanthopagrus latus*) is an ecologically significant marine fish species with a broad distribution across the Indo-West Pacific (Li et al., 2023). It is renowned for its delicious-tasting meat and high economic value, making it one of the most valuable economic fish species in the coastal regions of South China (Ahmed et al., 2022). Despite increasing production, yellowfin seabream ascites disease (YFSBAD) has emerged as a major threat to the industry for several years, primarily caused by infection with the scale drop disease virus (SDDV) (Fu et al., 2021). SDDV is a double-stranded DNA virus that induces clinical symptoms such as scale loss, fin rot, tail erosion, and can lead to mortality rates as high as 50% (De Groof et al., 2015). In 2020, Nurliyana et al. first reported that SDDV causes lethargy, severe scale loss, dorsal darkening, and ventral hemorrhage in intensively farmed Asian seabass in Malaysia (Nurliyana et al., 2020). Since then, SDDV has been subjected to next-generation sequencing, enhancing our understanding of its genetic composition (Kayansamruaj et al., 2020).

The rapid development of nucleic acid amplification technology has expanded its application in pathogen analysis. Several molecular detection methods for SDDV have been developed, including semi-nested PCR (Charoenwai et al., 2019), SYBR Green quantitative PCR (Sriisan et al., 2020), loop-mediated isothermal amplification (LAMP) (Dangtip et al., 2019) and conventional PCR (Senapin et al., 2019). However, these methods have inherent limitations; PCR and semi-nested PCR methods have the potential for false negatives in detecting viruses (Drosten et al., 2002). While LAMP allows for rapid qualitative detection, it does not provide quantitative analysis. SYBR Green quantitative PCR offers quantitative capabilities but necessitates the establishment of a standard curve. Droplet digital PCR (ddPCR) is an innovative absolute quantitative PCR technique characterized by high sensitivity, accuracy, and the ability to perform quantification without the need for a standard curve. It has been successfully applied in various fields, including liquid biopsy, noninvasive prenatal testing, pathogen detection, and genetically modified organism analysis (Lin et al., 2020; Hou et al., 2023), due to its advantages in sensitivity, precision, and reproducibility. In this study, we have developed a ddPCR method that is both sensitive and quantitative for the detection of SDDV. This approach will facilitate more effective disease monitoring and provide farmers with precise pathogen identification, thereby enhancing disease management strategies.

## 2 Materials and methods

### 2.1 Samples

A positive *Escherichia coli* plasmid was constructed using SDDV isolated from yellowfin seabream and then purified. A total of 30 clinical samples, including 20 diseased samples and 10 healthy yellowfin bream samples, were collected from farms located in Jinwan district, Zhuhai, China. The virus suspensions of infection spleen and kidney necrosis virus (ISKNV) and the virus suspensions of Mandarinfish Ranavirus (MRV) were provided by State Key Laboratory of Biocontrol, School of Life Science, Sun Yat sen University, Guangzhou, China. The fish sample infected with Tilapia Lake virus (TiLV) was obtained from Shenzhen Customs Animal and Plant Inspection and Quarantine Technology Center, Shenzhen, China. Samples from yellowfin seabream exhibiting signs of severe abdominal distension and near-terminal conditions post-SDDV challenge test were collected from various organs, including the liver, spleen, kidneys, intestines, brain, as well as blood, muscle, skin, and ascitic fluid. All the samples were stored at −80°C immediately after collection for later use.

### 2.2 Experimental instruments

Samples were subjected to qPCR assay using fluorescence quantitative PCR instrument (Real-time PCR thermal cycler qTOWER G, Analytik Jena, Germany). The samples were processed to generate microdroplets using drop maker instrument (Drop Maker M1, Targeting One, China). The generated microdroplets were amplified using rapid gradient PCR instrument (PCR A300, LongGene, China). The microdroplets were read using biochip analyzer (Chip Reader R1, Targeting One, China) to read the fluorescent microdroplet values. The data read by the biochip analyzer was analysed using ChipReader R1 software to derive the viral copy values.

### 2.3 Primer and probe design

According to published articles, the primers and probe was designed to target the SDDV *mcp* gene (NCBI gene bank accession no.: OM037668.1) (Fu et al., 2021) using Oligo 7.0 Software. The specificity of the primers and probe was examined by BLAST.^1^ The designed SDDV primer sequences did not have complementary sequences in the published yellowfin seabream genome (GeneBank ID: GCA_904848185.1) by BLAST analysis. The probe was labeled with carboxyfluorescein (FAM) as the fluorescent reporter and black hole quencher (BHQ1) as the fluorescence quencher. The primers and probe were synthesized by Sangon Biotech (Shanghai, China) Co., Ltd. and used in both ddPCR and qPCR (shown in Table 1).

**Table 1:** Primer and probe sequences for ddPCR-based quantification of SDDV.

		Sequence (5′-3′)	Product size (bp)
Primer	57-F	GCACTAATGATAATGCAATTTCTGTAC	109
	57-R	TCACGCTCTTCGTTGGTCAG	
Probe	57-P	CACCCGCTCTGACTGAAGTTAGTGTTATG	55

### 2.4 Tissues DNA extraction

The tissues sample DNA were extracted using MiniBEST Universal Genomic DNA Extraction Kit version 5.0 (#9765, TaKaRa, Japan). A 10 mg sample of liver, spleen, kidney, heart, intestine, brain, blood, muscle, skin and ascites of yellowfin seabream was taken and placed in a 2 mL centrifuge tube and cut into fine pieces. One hundred and eighty μL of buffer GL, 20 μL of proteinase K, and 10 μL of RNase A (10 mg/mL) were added, and placed in a warm bath in water at 56°C for 3 h until complete lysis. To the mixed lysate with samples, 200 μL of buffer GB and 200 μL of ethanol of 100% purity were added and mixed thoroughly. The Spin Column was placed on the Collection Tubu and the solution was pipetted into the Spin Column and centrifuged at 12,000 rpm for 2 min to remove the filtrate. Five hundred μL of Buffer WA was added to the Spin Column and centrifuged at 12,000 rpm for 1 min to remove the filtrate. Add 700 μL of Buffer WB along the inner wall of the centrifuge tube into the Spin Column and centrifuge at 12000 rpm for 1 min to remove the filtrate. Repeat the step of adding the Buffer WB. The Spin Column was placed on the Collection Tube and centrifuged at 12,000 rpm for 2 min. Place the Spin Column on a new 1.5 mL Centrifuge Tube, add 200 μL of Elution Buffer to the center of the Spin Column membrane, leave at room temperature for 5 min, and then centrifuge it at 12,000 rpm for 2 min to elute and obtain the genomic DNA of the sample, and stored at −20°C for spare use.

### 2.5 Virus DNA extraction

Virus DNA was extracted from ISKNV and MRV virus suspensions by using Viral DNA extraction Kit (#D3892-01, Omega Bio-tek, the United States). Two hundred and fifty μL of the virus sample was added to a centrifuge tube with 10 μL of OB Protease and 250 μL of Buffer BL (which contains 4 μL of Linear Acrylamide), vortexed and mixed for 15 s, and then incubated for 10 min at 65°C. To the mixture, 260 μL of anhydrous ethanol was added and vortexed and mixed at maximum speed for 20 s. The HiBind DNA Mini column was snapped into a 2 mL collection tube, then the lysate after addition of anhydrous ethanol and vortex mixing was transferred to the HiBind DNA Mini column and centrifuged at 8000xg for 1 min to remove the filtrate. Sleeve the HiBind DNA Mini column back into a new 2 mL collection tube, add 500 μL Buffer HB and centrifuge at 8000xg for 1 min to remove the filtrate. Slip the HiBind DNA Mini column back into a 2 mL collection tube, add 700 μL of DNA Wash Buffer, centrifuge at 8000xg for 1 min, and remove the filtrate. Sleeve the HiBind DNA Mini column back into a new 2 mL collection tube, add 700 μL DNA Wash Buffer for a second wash, centrifuge at 8000xg for 1 min, and remove the filtrate. Sleeve the HiBind DNA Mini column back into a 2 mL collection tube and centrifuge the empty column at maximum speed (15,000xg) for 2 min. Sleeve the HiBind DNA Mini column into a new 1.5 mL centrifuge tube, add 100 μL of pre-warmed Elution Buffer at 65°C, let stand at room temperature for 5 min, and 8,000xg The DNA was eluted by centrifugation for 1 min. The HiBind DNA Mini column was inserted into a new 1.5 mL centrifuge tube, and a second elution was performed using Elution Buffer, and the eluted DNA was stored at −20°C for spare use.

### 2.6 Virus RNA extraction

Virus RNA was extracted from NNV and TiLV infected tissues by using MiniBEST Universal RNA Extraction Kit (#9767, TaKaRa, Japan). The cryo-frozen 20 mg viral tissue sample was quickly added to a 1.5 mL sterilized centrifuge tube, 350 μL of lysis Buffer RL (which includes DTT Solution) was added, and the tissue was broken using a tissue breaker, and blown repeatedly using a pipette until there was no obvious precipitate in the lysate. The lysate was centrifuged at 12,000 rpm for 5 min at 4°C. Aspirate the supernatant into a new 1.5 mL RNase Free Tube. Place the gDNA Eraser Spin Column onto a 2 mL Collection Tube and transfer the supernatant into the gDNA Eraser Spin Column and centrifuge at 12,000 rpm for 1 min. Remove the gDNA Eraser Spin Column and retain the filtrate in the 2 mL centrifuge tube. Add 2 mL of 70% ethanol to the above filtrate and mix the solution well using a pipette. Immediately transfer all of the mixture into the RNA Spin Column and centrifuge at 12,000 rpm for 1 min, remove the filtrate and place the RNA Spin Column back into the 2 mL Collection Tube. Five hundred μL of Buffer RWA was added to the RNA Spin Column, centrifuged at 12,000 rpm for 30 s to remove the filtrate, and then 600 μL of Buffer RWB was added along the inside wall of the RNA Spin Column centrifuge tube. Genomic DNA was removed using DNase I. The RNA Spin that had removed genomic DNA was removed. Column was repositioned on a 2 mL Collection Tube and centrifuged at 12,000 rpm for 2 min. The RNA Spin Column was placed on a 1.5 mL RNase Free Collection Tube, and 200 μL of RNase was added to the center of the RNA Spin Column membrane. Free dH_2_O at the center of the RNA Spin Column membrane, let it stand at room temperature for 5 min, and centrifuge at 12,000 rpm for 2 min to elute the RNA.

The total volume of the ddPCR reaction solution was 30 μL, including 29 μL of premix for the ddPCR probe assay and 1 μL of PCR reaction template extracted from the samples to be tested. ddPCR reaction liquid system consisted of 15 μL of mix (2×), 2.4 μL of upstream primer 57-F (10 μM), 2.4 μL of downstream primer 57-R (10 μM), 0.75 μL of probe 570P, and 1 μL of PCR reaction template (concentration less than 50 ng/μL), and then 8.45 μL of RNAase-free distilled water was added to 30 μL. The primer concentration and probe concentration were both 10 μM. PCR amplification reaction conditions were as follows: pre-denaturation at 95°C for 10 min; denaturation at 95°C for 30 s, annealing at 63°C for 1 min, 39 cycles, and a rate of temperature change of 1.5°C/s. The Universal Kit for Microdroplet Assay was purchased from Beijing TargetingOne Biotechnology Co., Ltd. (#10002), Beijing, China.

### 2.7 Samples from challenge test DNA extraction

Animal Tissue Genomic DNA Extraction Kit from Beijing Dingguo Changsheng Biotechnology Company Limited was used to extract DNA from samples collected in the challenge test. The samples obtained were cut into small pieces with scissors. Six hundred μL of Lysis Buffer was added, AND mixed thoroughly. Then left to stand at room temperature for 5 min. After that, 10 μL of Proteinase K was added, and mixed well, then water-bathed for 1 min at 56°C. The samples were then extracted into small pieces with scissors, added to the Lysis Buffer, mixed well, and then water-bathed for 1 min at 56°C. hours until the tissue was completely lysed. Centrifuge at 12,000 rpm for 10 min, collect the supernatant into a new centrifuge tube, add 800 μL of anhydrous ethanol and mix well. The mixture was transferred to a centrifuge column and centrifuged at 12,000 rpm for 1 min to remove the waste liquid. Add 700 μL of Wash buffer A containing anhydrous ethanol and centrifuge at 12000 rpm for 1 min to remove the waste solution. Add 500 μL of Wash buffer B containing anhydrous ethanol and centrifuge at 12000 rpm for 1 min to remove the waste solution. Add 500 μL of Wash buffer B and centrifuge at 12000 rpm for 1 min to remove the waste solution. Centrifuge again at 12,000 rpm for 2 min, place the column in a new centrifuge tube, open the column lid and prevent it from evaporating in a 37°C thermostat for 5 min until the ethanol is completely evaporated. Add 200 μL of TE buffer at 60°C in the center of the silica matrix membrane, leave it at room temperature for 5 min, and centrifuge at 12000 rpm for 30 s. Transfer the solution obtained by centrifugation to the centrifugation column again, leave it at room temperature for 2 min, and centrifuge at 12000 rpm for 2 min, and the solution obtained is the purified genomic DNA.

### 2.8 Optimization of reaction conditions for the ddPCR method

Threshold line, annealing temperature are the key factors affecting the results and sensitivity of ddPCR assay. The PCR reaction template extracted from the SDDV samples to be tested was used as a reference and the reaction conditions were optimized, and the copy number of the ddPCR results was compared to select the final optimized conditions.

### 2.9 Sensitivity test of the ddPCR method

The SDDV positive plasmid with a concentration of 2.3 × 10^9^ copies/μL was subjected to gradient dilutions of 10,100,1,000, 10,000 and 100,000 to reach viral concentrations below the upper limit of the ddPCR method detection, and then subjected to ddPCR detection test. The ddPCR results were used to establish a linear plot, and the sensitivity was calculated by linear dynamic range.

### 2.10 Sensitivity and accuracy comparison of ddPCR and qPCR

The SDDV positive plasmid was diluted (refer to section 2.4) and detected using ddPCR and qPCR, respectively, and the sensitivity and accuracy of the two assays were analysed by comparing copy numbers and amplification curves.

### 2.11 Reproducibility validation of the ddPCR method

Ten replicates of 1 μL from the same sample were taken, and these 10 replicates were tested using the ddPCR method, and the reproducibility of the method was verified by calculating the relative standard deviation (RSD) of copy number. The calculation methods were as follows,


$$\mathrm{Mean}=\overset{n}{\underset{i=1}{\sum }}\mathrm{Ci}/n$$

RSD=SD/Mean×100%

Where $\overset{n}{\underset{i=1}{\sum }}\mathrm{Ci}$ /n denotes the sum of concentration measurements from all the experiments, n denotes the total number of experiments, and SD denotes the standard deviation of all concentration measurement.

### 2.12 Challenge test

The SDDV solution used in the challenge test was obtained from the State Key Laboratory of Biocontrol, School of Life Sciences, Sun Yat-sen University, Guangzhou, China. Twenty yellowfin seabreams without SDDV were purchased from a local farm in Jinwan District, Zhuhai, China, and were 12–14 cm in length. The yellowfin seabream was firstly placed in indoor fiberglass buckets for 2 weeks of temporary rearing, during which time the yellowfin seabream were fed with commercial diets. After temporary rearing, the yellowfin seabream was anesthetized with eugenol, and 3 × 10^8^ TU/mL of SDDV diluted by sterile PBS was injected intraperitoneally in a volume of 100 μL per fish, and the control group was injected with the same volume of PBS. The challenge test lasted for 7 days without feeding.

### 2.13 Practical application of the clinical samples

Liver, spleen, kidney, heart, gut, brain, blood, muscle, skin and ascites samples were collected from fish that had performed the SDDV challenge test and had obvious pathological manifestations, and these samples were tested using the established ddPCR method.

## 3 Results

### 3.1 Threshold line determination

**Figure 1:**
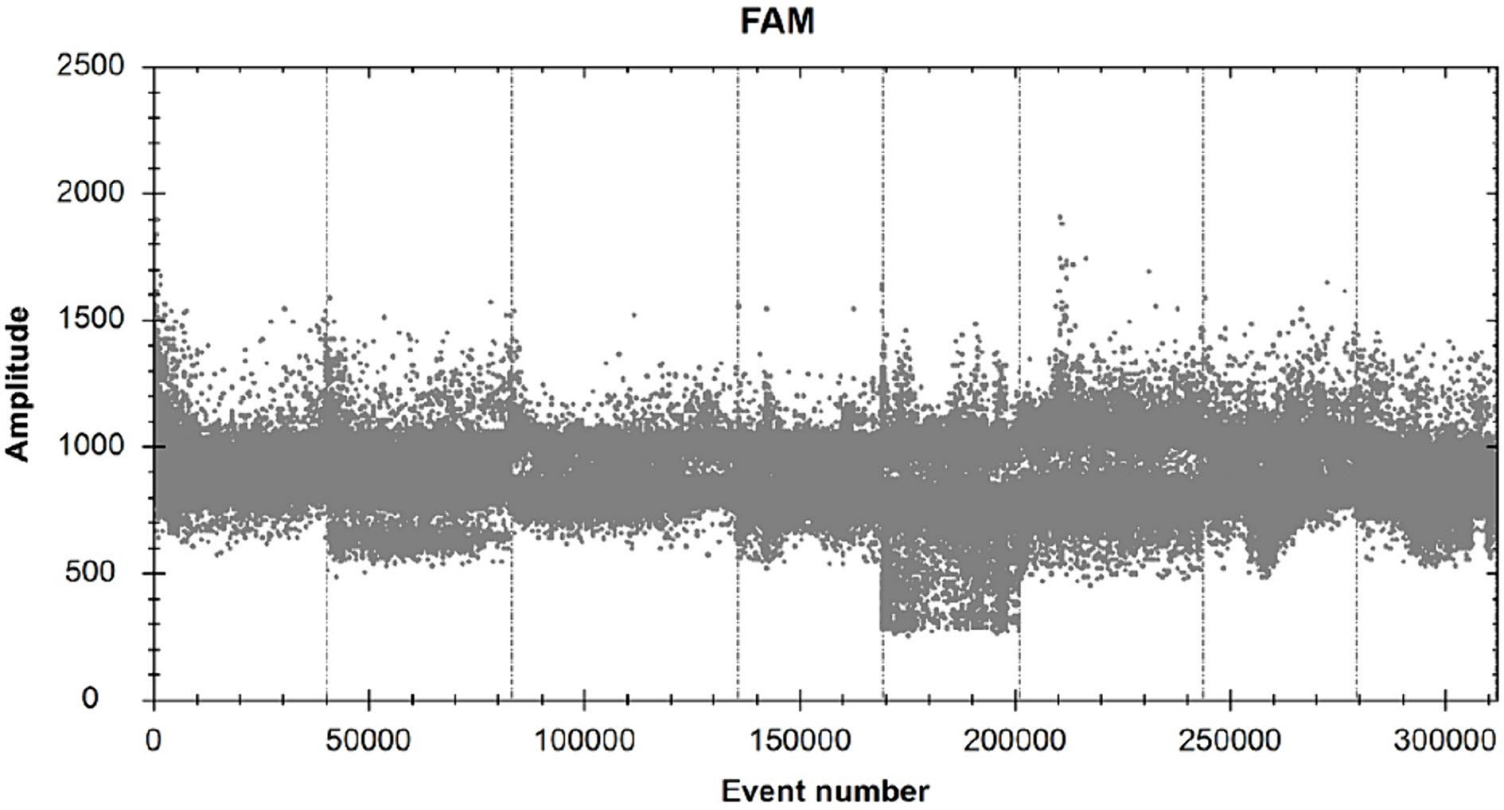
FAM scatter plot of RNAase-free distilled water.

All reaction steps are performed with reference to “The Digital MIQE Guidelines Update: Minimum Information for Publication of Quantitative Digital PCR Experiments for 2020” (Group and Huggett, 2020). According to the ddPCR reaction solution configuration system composition and amplification reaction described herein, 1 μL of RNAase-free distilled water was substituted for 1 μL of PCR reaction template to make a blank sample reaction system, amplification was carried out, and microdroplets were detected by using a microdroplet analyzer for readout to analyze the number of fluorescent microdroplets emitting fluorescence in each tube of the reaction system (Figure 1). By performing ddPCR on 10 blank samples, the threshold line position was determined to be 1900.

### 3.2 Annealing temperature optimization

**Figure 2:**
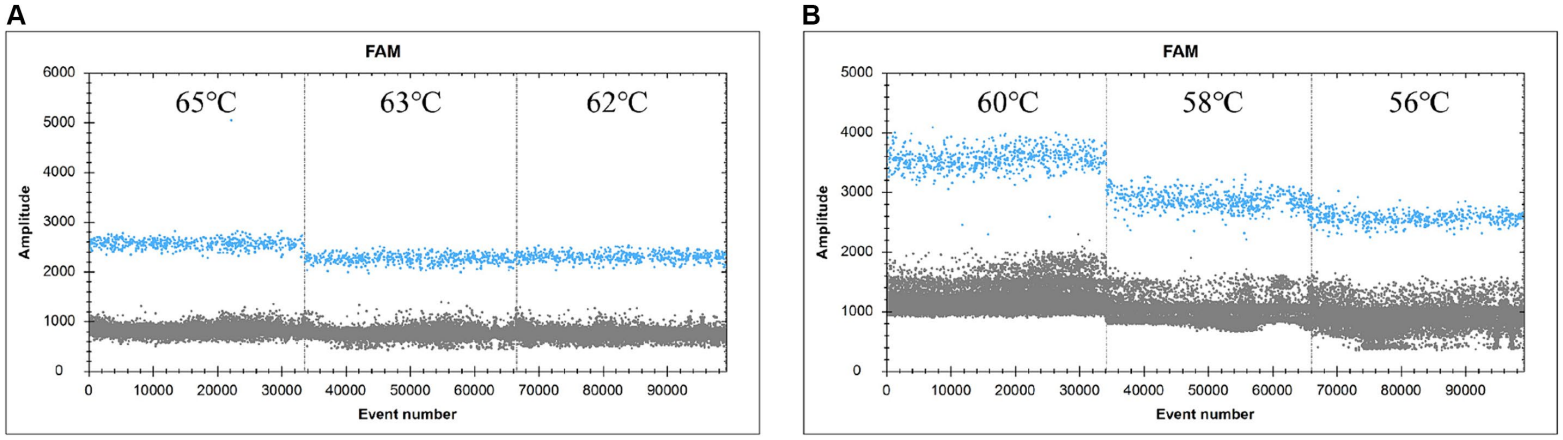
FAM scatter plot under different annealing temperature conditions. (**A** and **B**) simply indicate the temperature from high to low.

The ddPCR reaction was run with the primers and probe concentrations determined above (Figure 2). The annealing temperature was the independent variable in this experiment, and a total of six annealing temperatures were set, namely 65.0°C, 63.0°C, 62.0°C, 60.0°C, 58.0°C, and 56.0°C, which corresponded to the number of copies of 980 copies, 1,018 copies, 1,080 copies, 1,228 copies, 1,088 copies, 1,582 copies, 1,338 copies (heteroband), and 1,338 copies (heteroband), respectively. Therefore, the optimal annealing temperature for this assay is 60°C.

### 3.3 Specificity validation of primers and probes by ddPCR method

**Figure 3:**
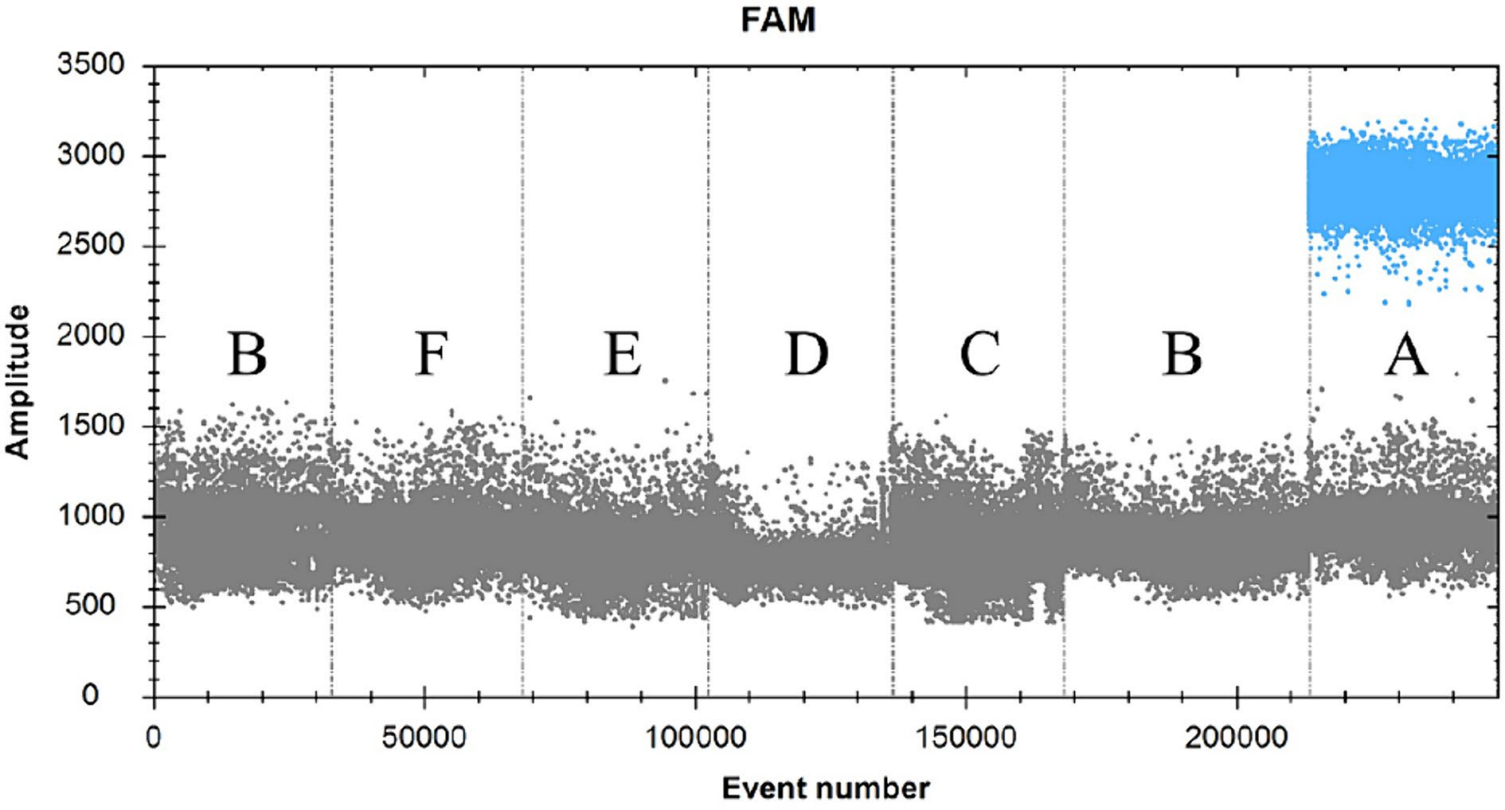
FAM scatter plot of ddPCR validation for different viruses.

SDDV-positive plasmid was used as a positive control (A), RNAase-free distilled water was used as a negative control (B), and ISKNV (C), MRV (D), NNV (E), and TiLV (F) were used as viral templates to be tested (Figure 3; Table 2). The results showed that only group A could detect the signal, and the rest of the groups had no signal, indicating that the primers and probes in this experiment had good specificity.

**Table 2:** ddPCR specificity validation for different viruses.

Virus	SDDV-positive plasmid	RNAase-free distilled water	ISKNV	MRV	NNV	TiLV
Results	Positive	Negative	Negative	Negative	Negative	Negative

### 3.4 Sensitivity test of the ddPCR method

**Figure 4:**
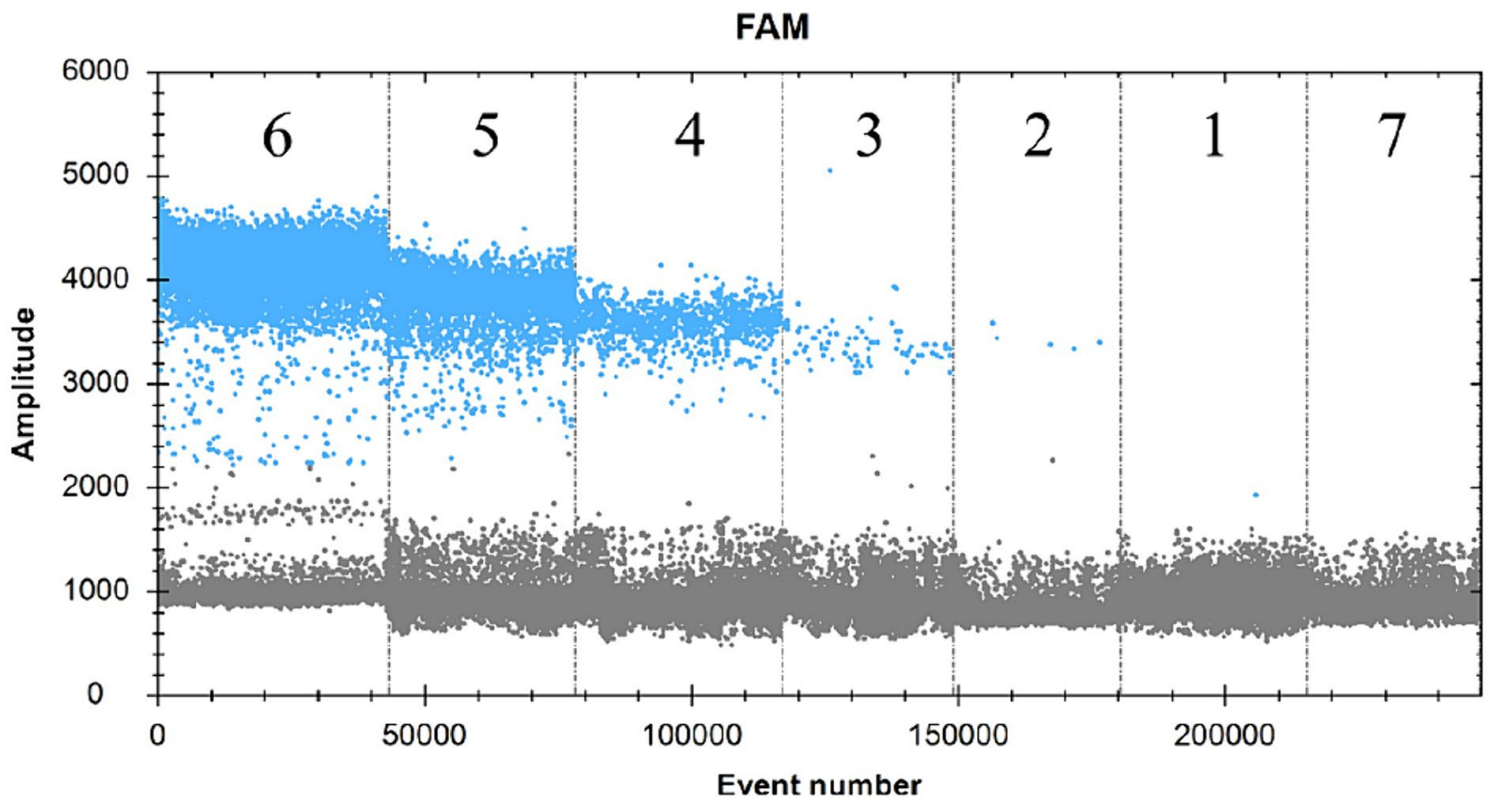
FAM scatter plot of ddPCR method sensitivity test.

The SDDV virus-positive plasmid was subjected to a 10-fold gradient of serial dilutions of 10,100,1,000,10,000 and 100,000. A total of six concentration gradients were set up at 1.37 × 10^5^, 1.37 × 10^4^, 1.37 × 10^3^, 1.37 × 10^2^, 1.37 × 10^1^, and 1.37 × 10^0^ copies/μL, which were established in the text according to the ddPCR assay. The results showed that interval 1 was 1.2 copies/μL, interval 2 was 30.5 copies/μL, interval 3 was 386.7 copies/μL, interval 4 was 3,619.3 copies/μL, interval 5 was 34,894.2 copies/μL, interval 6 was 2,967,717.3 copies/μL, and there was no fluorescence signal in interval 7 (negative control) by direct calculation of Poisson distribution (Figure 4). Therefore, the lowest detection limit of this assay for SDDV was 1.4–1.7 copies/μL.

### 3.5 Sensitivity and accuracy comparison of ddPCR and qPCR methods

In this experiment, the plasmid containing the SDDV genomic fragment was diluted 10, 100, 1,000, 10,000 and 100,000 times by sterile PBS (pH 7.2), and its copy number/amplification curve was detected by ddPCR and qPCR, respectively. The results showed that when the plasmid was diluted 10 times, the ddPCR detection result was fully positive, indicating that the plasmid concentration was too high and had exceeded the maximum threshold for its detection (Table 3). Both ddPCR and qPCR could accurately detect SDDV when the dilution was 100, while when the plasmid dilution was 100,000, the CT value of qPCR method had exceeded 35, which could not effectively detect the SDDV content in the samples, while the ddPCR method could still accurately detect the copy number of SDDV in the samples. Therefore, when detecting low concentration of SDDV by ddPCR and qPCR, if the copy number of SDDV is low, the qPCR method cannot effectively detect SDDV in the sample, indicating that ddPCR is more sensitive than qPCR in the detection of SDDV.

**Table 3:** Sensitivity and accuracy comparison of ddPCR and qPCR methods.

qPCR detection results		ddPCR detection results	
Plasmid concentration	Average CT value	Plasmid concentration	Actual copies
1.37 × 10^5^	20.55	1.37 × 10^5^	296717.3
1.37 × 10^4^	23.22	1.37 × 10^4^	34894.2
1.37 × 10^3^	26.44	1.37 × 10^3^	3619.3
1.37 × 10^2^	30.22	1.37 × 10^2^	386.7
1.37 × 10^1^	35.95	1.37 × 10^1^	30.5
1.37 × 10^0^	/	1.37 × 10^0^	1.2

### 3.6 Reproducibility validation of the ddPCR method

The same set of samples was chosen for all validations. This sample was repeated 10 times with 1 μL each time and was tested using ddPCR technique and the RSD was calculated to be 4.37%. The results proved that the method was reproducible and stable and reliable results could be obtained (Table 4).

**Table 4:** Reproducibility validation of the ddPCR method.

Replicates	Copies (×10^3^ copies/μL)	Average (×10^3^ copies/μL)	Standard Deviation	RSD/%
1	3.52	3.74	0.16	4.37%
2	3.51			
3	3.62			
4	3.96			
5	3.70			
6	3.93			
7	3.87			
8	3.82			
9	3.79			
10	3.63			

### 3.7 Practical application of the clinical samples

**Figure 5:**
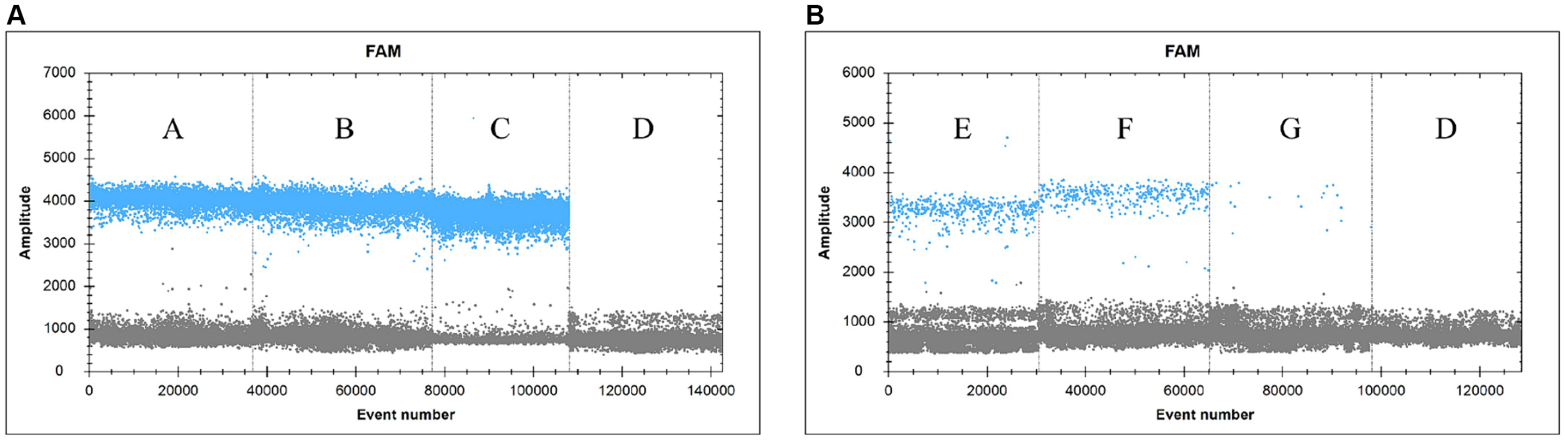
Practical application of the clinical samples. (**A**: Liver, **B**: Spleen, **C**: Kidney, **D**: sterile PBS (negative control), **E**: blood, **F**: ascites, **G**: intestine).

Samples were collected from dying yellowfin seabream after SDDV challenge test and were subjected to tissue DNA extraction for detection of viral load using the ddPCR assay technique described in this study (Figure 5). The results showed that the ddPCR method detected high levels of virus in liver (A), spleen (B) and kidney (C), low levels of virus in blood (E), ascites (F) and intestine (G), and no result was found in negative control (D). The copy numbers of SDDV were 17,963.7 copies/μL in liver, 27,025.4 copies/μL in spleen, 178,954.3 copies/μL in kidney, 1,012.0 copies/μL in blood, 747.0 copies/μL in ascites, and 38.0 copies/μL in intestine by the ddPCR method, respectively.

## 4 Discussion

The prevalence of SDDV infection is high and its outbreaks can cause significant economic losses to the aquaculture industry, in addition, the costs associated with disease management, including treatment, diagnosis, and disposal of infected fish, further increase the cost of farming. Infected fish exhibit reduced growth rates, reduced feed utilization, and an impaired state of health that hinders normal production (Kiat et al., 2023). In addition, SDDV is highly transmissible, and the escape of fish carrying SDDV can lead to environmental damage and loss of biodiversity in new ecosystems, seriously affecting the export potential of the local aquaculture industry (Ji et al., 2023).The ddPCR method is now widely used for specific and sensitive detection of various types of viruses, such as ISKNV in *Siniperca chuatsi* (Lin et al., 2020), ranavirus in *Micropterus nigricans* (Jiang et al., 2023), herpesvirus in *Carassius auratus* (Zhao et al., 2024) and parvovirus in *Oreochromis mossambicus* (Zhao et al., 2023). In this study, we developed a ddPCR method to detect and quantify SDDV viral vectors, which showed high sensitivity and convenience.

Virus detection techniques have gradually gained attention from the end of the 20th century. Detection techniques for SDDV are also diverse, but each has its own advantages and disadvantages. Although semi-nested PCR has been an effective means of nucleic acid amplification and detection, it still has several drawbacks, such as low detection accuracy, which prevents effective detection of target samples with low sequence concentration, as well as long detection time and cumbersome steps, which are not conducive to the application in practical production. Charoenwai et al. (2019) utilized a modified semi-nested PCR method for the detection of SDDV in Asian sea bass, but the sensitivity was poor with a lower limit of detection of 100copies/μL, which was much higher than the lower limit of detection of the assay utilized for ddPCR in this study, 1.4–1.7 copies/μL. This makes the semi-nested PCR method unable to detect SDDV carried by fish in time for proper disease prevention and control. Li et al. also developed a ddPCR method for the detection of ISKNV with a detection limit of 1.5copies/μL, which is much lower than that of TaqMan real-time PCR with a detection limit of 34copies/μL, confirming that the ddPCR method has the advantage of detection in the samples with low amount of ISKNV viral vectors, which is beneficial for the monitoring of the source as well as the transmission route of ISKNV (Lin et al., 2020). Rungrueng et al. (2021) added 10 mM ammonium sulphate to the PCR reaction buffer based on Charoenwai et al.’s method, which effectively improved the detection sensitivity and avoided false-negative results, but the sensitivity still fell short of the ddPCR. When human parechoviruses were detected by the semi-nested PCR method, the limit of detection was 10 RNA copies, whereas for human hantaviruses detection, the limit of detection was as high as 100 RNA copies (Nunes et al., 2019), which still did not reach the sensitivity of the method studied in this experiment (Nix et al., 2010).

SYBR Green quantitative PCR method is a globally recognized molecular biology technique for virus detection, which is mature and specific enough to detect a large range of viral vectors (Olveira et al., 2021). However, SYBR Green quantitative PCR experiments have more steps and require standard curves for quantification, which have certain operational limitations in fisheries (Giglioti et al., 2022). The ddPCR method in this study has high sensitivity for detecting samples with low viral vectors, which can match the practical application scenario in aquaculture disease detection. In contrast, primers with high specificity were provided in this study, which highly guaranteed the reliability and accuracy of the results. Similar problems also exist with the LAMP method, which involves the participation of multiple primers, but can still lead to false-positive results when non-specific DNAs are similar to the target sequence or polymorphisms exists (Rungrueng et al., 2021), in addition to the slow amplification rate of LAMP and the possibility of primer interferences (Sukonta et al., 2022).

PCR methods, on the other hand, can only provide relative quantitative results, not absolute quantitative results (Al-Bayati et al., 2023; Mijač et al., 2023), and the detection of low levels of target sequences can lead to false-negative results (Pecoraro et al., 2022), and the experimental procedure is cumbersome and not very suitable for immediate detection (Ruijter et al., 2021). Even different sources of Taq DNA polymerase can lead to differences in experimental results (Rungrueng et al., 2021). The ddPCR method is one of the PCR methods, in which the well-mixed template is separated into a number of small droplets, and each droplet reacts in the PCR system and passes through the detector one by one, and the copy number of the target virus can be directly calculated according to the ratio of positive and negative microdroplets, thus realizing the absolute quantification of the target virus. Compared with the qPCR method, the ddPCR method does not require nucleic acid standards or the establishment of a standard curve, and allows for direct absolute quantification with the simultaneous advantages of qPCR. However, the upper detection limit of ddPCR is not as reliable as that of qPCR, and it is necessary to dilute the samples with high concentration to below the upper detection limit of ddPCR (Henrich et al., 2012). It is assumed that the reason for this result is due to the difference in the reading process of fluorescent signals between the two methods. ddPCR reads the fluorescent signals in the microdroplet at the end point of the reaction, while qPCR monitors the changes in fluorescent signals in real time, but the principle of the two detection methods is the same.

In this research, the results of SDDV can be used to determine the viral content of target sample directly by the ddPCR method, based on the copy number of nucleic acid, eliminating the need to set up a standard curve and further simplifying the operational procedures.

## 5 Conclusion

In conclusion, a novel ddPCR method for the detection of SDDV was established in this study. Compared with other PCR methods, ddPCR method has high sensitivity and specificity, and does not require a standard curve and has simple detection steps. It is important for the prevention and monitoring of SDDV in aquaculture.

## Data Availability

The datasets presented in this study can be found in online repositories. The names of the repository/repositories and accession number(s) can be found in the article/supplementary material.

## Footnotes

^1^https://www.ncbi.nlm.nih.gov/tools/primer-blast/
